# Impaired sleep resilience underlies transient neural instability in insomnia disorder

**DOI:** 10.1016/j.isci.2026.116151

**Published:** 2026-06-04

**Authors:** Chao Yang, Hai Gu, He Chen, Guohua Chen, Xinhua Song, Xin Liu, Qing Yang, Guang Wang, Cancheng Li, Heng Gu, Shaodi Wang, Peiyang Guo, Jiawei Zhao, Lu Wang, Rui Wang, Changming Wang, Junhua Mei, Xiaoli Li

**Affiliations:** 1 State Key Laboratory of Cognitive Neuroscience and Learning & IDG/McGovern, Beijing Normal University, Beijing 100875, China; 2 School of Automation Science and Engineering, South China University of Technology, Guangzhou 511442, China; 3 Department of Neurology, Wuhan First Hospital, Wuhan 430022, China; 4 Clinical College of Traditional Chinese Medicine, Hubei University of Chinese Medicine, Wuhan 430065, China; 5 School of Biological Science and Medical Engineering, Beihang University, Beijing 100191, China; 6 Ningbo Institute of Dalian University of Technology, Ningbo 315014, China; 7 Department of Anesthesiology, Peking University Third Hospital, Beijing 100191, China; 8 Beijing Luhe Hospital, Capital Medical University, Beijing 101149, China; 9 College of Biomedical Engineering, Capital Medical University, Beijing 100069, China; 10 Jingtong Brain Research Laboratory, Beijing 101100, China

**Keywords:** Health sciences, Medicine, Neurology

## Abstract

This study quantified sleep resilience in insomnia disorder (ID) by examining the brain’s ability to resist, contain, and recover from auditory perturbations during nocturnal sleep. 58 ID patients and 43 healthy controls underwent real-time staged nocturnal recordings with event-locked auditory stimulation. Neural responses were evaluated using evoked complexity, P300, event-related phase-amplitude coupling (ERPAC), and coupling pattern diversity. Compared with controls, ID patients showed elevated evoked complexity and enlarged P300 responses, especially in N3 sleep, indicating reduced resistance to disturbance. ID patients also exhibited stronger fronto-parietal ERPAC during the middle post-stimulus window, suggesting impaired containment. These abnormalities weakened in the late window, consistent with relatively preserved recovery. In conclusion, ID is marked by impaired sleep resilience, particularly in deep sleep, and sleep resilience may provide useful objective markers of nocturnal instability.

## Highlights

• Sleep resilience is impaired in insomnia during deep N3 sleep
• Auditory perturbations increase neural complexity across sleep stages
• Frontal P300 amplification indicates heightened neural responsiveness to deviant stimuli
• Short-term fronto-parietal θ-β coupling indicates transient containment deficits

## Graphical abstract

**Figure:**
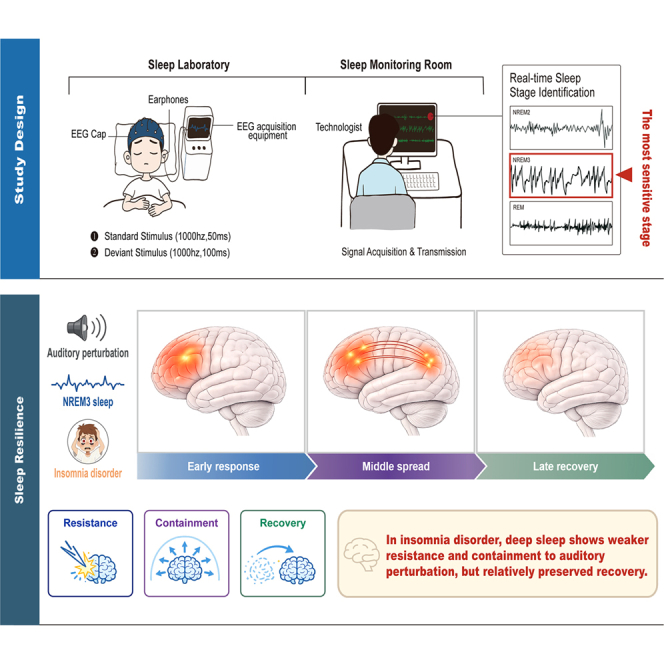

## Introduction

Insomnia disorder (ID) ranks among the most prevalent and burdensome sleep conditions worldwide. Chronic insomnia is closely associated with elevated cardiovascular and neuropsychiatric risks, as well as diminished quality of life. However, its clinical diagnosis still relies primarily on subjective questionnaires, lacking quantifiable and replicable objective biomarkers.^1^^,^^2^ Although polysomnography (PSG) can characterize the macroscopic sleep architecture,^3^ conventional resting-state or whole-night averaged metrics often fail to capture the transient susceptibility to external perturbations and the underlying network instability during sleep.^4^^,^^5^^,^^6^ Introducing high-temporal-resolution, event-locked external stimuli during nocturnal sleep and characterizing the brain’s phasic responses have emerged as a promising pathway to elucidate the neuropathology of ID and establish objective biomarkers.^7^^,^^8^

Prevailing pathophysiological models posit that the core feature of ID is chronic hyperarousal spanning the cognitive-emotional, autonomic nervous, and cortical domains.^9^ Objective evidence indicates that even during sleep, individuals with ID exhibit enhanced high-frequency electroencephalogram (EEG) activity and sympathetic activation, supporting a trait-like, persistent hyperarousal state.^10^ However, debate persists regarding whether this state is one of sustained elevations or manifests as phasic intrusions of “wake-like activity” into sleep.^11^ Quantitative EEG studies have revealed increased fast-frequency (beta/gamma [β/γ]) power and decreased slow-wave power during non-rapid eye movement (NREM) sleep in ID, suggesting global cortical activation.^12^^,^^13^ Sleep microstructure findings likewise suggest increased instability and brief wake-like intrusions in ID.^14^ These findings suggest that alongside global hyperarousal, ID may involve brief, bursting intrusions of wake-like activity. As resting-state or whole-night averaging approaches smooth out transient event-related responses, previous research has provided limited insight into the critical processes underlying how sleep, particularly deep sleep, becomes destabilized under perturbation.

Beyond global spectral features, the coordination of cross-frequency interactions may represent a crucial dimension for maintaining sleep stability. Cross-frequency coupling (CFC), by which faster rhythms’ amplitude is regulated by the phase of slower oscillations, acts as a key neural information-processing mechanism.^15^ CFC is thought to support sensory gating and information processing across brain states. Nevertheless, evidence for such coupling dysregulation in ID remains scarce. Previous studies have predominantly focused on isolated band powers, while phase-amplitude coupling (PAC), a metric for sleep integrity, has been insufficiently explored. Given the difficulty of directly assessing high-frequency-related coupling during deep sleep, theta (θ)-phase to β-amplitude coupling may provide a practical readout of cross-frequency coordination relevant to sleep gating in ID.^16^^,^^17^ Furthermore, post-stimulus signal complexity provides a complementary perspective on the orderliness, predictability, and stability of neural responses under perturbation.

Building upon this conceptual framework, the present study adopts a sleep resilience approach^18^^,^^19^ to operationalize the brain’s capacity to resist and adapt to perturbations during sleep along three complementary dimensions: disturbance resistance, containment, and recovery.^20^^,^^21^ Disturbance resistance refers to minimizing the initial deviation induced by perturbation, containment to constraining the spread, rigidification of evoked activity while preserving dynamic flexibility, and recovery to the rapid return toward the pre-perturbation state. Although these dimensions unfold continuously across the trial, containment-related abnormalities were hypothesized to be most evident in the middle post-stimulus window, whereas recovery was primarily evaluated in the late window. Disturbance resistance was indexed using complementary measures that captured partly overlapping phases of the perturbation response, including the overall post-stimulus complexity profile reflected by permutation entropy (PE)^22^ and the evaluative response indexed by frontal P300. To characterize these processes, the present study employed an event-locked auditory perturbation paradigm with real-time manual sleep staging, and combined PE, frontal P300, θ-β event-related phase-amplitude coupling (ERPAC), and Lempel-Ziv complexity (LZC) of coupling patterns as complementary neurophysiological measures of sleep resilience.

Guided by this rationale, we posited that ID would be associated with compromised sleep resilience in response to exogenous auditory disturbances during nighttime sleep, especially during the deep sleep stage (N3), manifesting as: (1) reduced disturbance resistance, reflected by elevated post-stimulus neural complexity indexed by PE and an amplified evaluative response indexed by frontal P300 amplitude; (2) insufficient containment, characterized by abnormally enhanced θ-β cross-frequency coordination along fronto-parietal pathways, indexed by ERPAC, alongside convergent and less diverse coupling patterns indexed by LZC; and (3) largely intact recovery, indicated by a normalization of CFC strength and coupling pattern complexity in later post-stimulus windows, such that ERPAC and LZC in ID become comparable to those of healthy controls, suggesting that the abnormalities are transient and confined to the early processing stage. Furthermore, we pre-specified exploratory mediation analyses to evaluate whether the affective symptom burden mediates the relationship between nocturnal CFC phenotypes and insomnia severity, thereby providing an initial quantification of the link between neurophysiological phenotypes and clinical symptoms. Collectively, this study aims to clarify how perturbation-related neural responses during sleep may reveal objective markers of deep sleep instability in ID.

## Results

### Cohort and clinical baseline disparities

Baseline assessments were first conducted to verify group comparability: gender distribution was analyzed via chi-square test, showing no significant difference (*p* > 0.05); body mass index (BMI) also had no statistically significant disparity between the groups (*p* > 0.05). As detailed in Table 1, significant differences emerged across multiple clinical scales: compared to healthy controls (HCs), the ID group scored lower on the mini-mental state examination (MMSE; *p* < 0.001), indicating poorer overall cognitive ability; scored higher on both the Hamilton Anxiety Rating Scale (HAMA) and Hamilton Depression Rating Scale (HAMD) (both *p* < 0.001), reflecting more severe anxiety and depressive symptoms; and scored higher on the Insomnia Severity Index (ISI) and Pittsburgh Sleep Quality Index (PSQI) (both *p* < 0.001), signifying more prominent sleep disturbances. Collectively, these results confirmed that the ID group exhibited comprehensive impairments in cognitive function, emotional regulation, and sleep quality relative to HCs. Given the substantial difficulty in nighttime data collection, complete data for specific sleep stages could not be obtained from some participants. Thus, the actual sample sizes for each sleep stage (HC, ID) were as follows: non-rapid eye movement stage 2 (N2) (38, 53), non-rapid eye movement stage 3 (N3) (27, 40), and rapid eye movement (REM) (33, 35).

**Table 1:** Clinical baseline disparities between groups

	HC	ID	t/χ^2^	*p* value
Participants (M, F)	43 (16, 27)	58 (14, 44)	2.02	0.155
Age, years, mean (SD.)	40.81 (13.88)	45.71 (13.12)	−1.81	0.074
Clinical scale, mean (SD)				
BMI	22.87 (2.70)	22.67 (3.74)	0.30	0.767
MMSE	29.67 (0.68)	28.22 (2.11)	4.90	<0.001
HAMA	4.79 (3.83)	19.74 (5.44)	−16.20	<0.001
HAMD	4.28 (3.84)	18.34 (6.15)	−14.10	<0.001
ISI	3.40 (2.14)	13.95 (5.57)	−13.18	<0.001
PSQI	4.70 (2.19)	14.78 (4.92)	−13.86	<0.001

### Disturbance resistance: Evoked complexity and evaluative response

**Figure 1:**
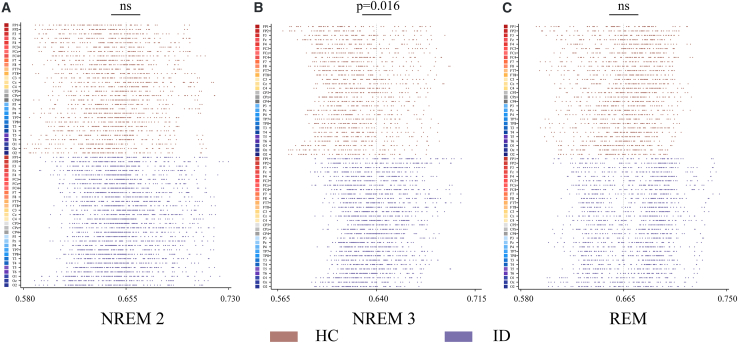
Comparison of evoked neural complexity between HC and ID groups across different sleep stages (A) NREM2: No significant difference in PE was observed between the HC and ID groups (HC − ID = −0.007, 95% CI [−0.016, 0.002], *t* = −1.43, *p* = 0.153, p_FDR = 0.230). (B) NREM3: PE was significantly higher in the ID group than in HCs (ID: 0.64 ± 0.02; HC: 0.62 ± 0.02; HC − ID = −0.013, 95% CI [−0.024, −0.002], *t* = −2.43, *p* = 0.016, p_FDR = 0.048, Cohen’s d = −0.61). (C) REM: No significant between-group difference in PE was detected (HC − ID = −0.006, 95% CI [−0.017, 0.004], *t* = −1.17, *p* = 0.245, p_FDR = 0.245). The HC group is represented in russet, and the ID group is represented in purple. The PE was calculated from EEG data within the 0–800 ms post-stimulus window, with standard and deviant trials combined. ns, not significant. p_FDR denotes the Benjamini-Hochberg false discovery rate-adjusted *p* value.

To assess neural responses to external disturbance during sleep, PE was calculated within the 0–800 ms post-stimulus window, with standard and deviant trials pooled to characterize the overall temporal complexity of neural activity under auditory stimulation. Linear mixed-effects model analyses revealed a significant main effect of group (F(1, 226) = 8.81, *p* = 0.003, partial eta-squared (η_p_^2^) = 0.038), indicating that individuals with ID exhibited higher overall PE than HCs. A significant main effect of sleep stage was also observed (F(2, 226) = 51.89, *p* < 0.001, η_p_^2^ = 0.315), with PE showing a systematic decrease as sleep depth increased. The group × sleep stage interaction was not significant (F(2, 226) = 0.57, *p* = 0.568, η_p_^2^ = 0.005). Planned post hoc comparisons further demonstrated that group differences were the most pronounced during deep sleep. Specifically, in N3 sleep, PE was significantly higher in the ID group than in HCs (ID: 0.64 ± 0.02; HC: 0.62 ± 0.02; HC − ID = −0.013, 95% CI [−0.024, −0.002], *t* = −2.43, *p* = 0.016, p_FDR [false discovery rate] = 0.048, Cohen’s d = −0.61, Figure 1B), indicating greater evoked temporal complexity during auditory stimulation in ID at this stage. In contrast, no significant group differences were observed in N2 sleep (HC − ID = −0.007, 95% CI [−0.016, 0.002], *t* = −1.43, *p* = 0.153, p_FDR = 0.230, Figure 1A) or REM sleep (HC − ID = −0.006, 95% CI [−0.017, 0.004], *t* = −1.17, *p* = 0.245, p_FDR = 0.245, Figure 1C).

**Figure 2:**
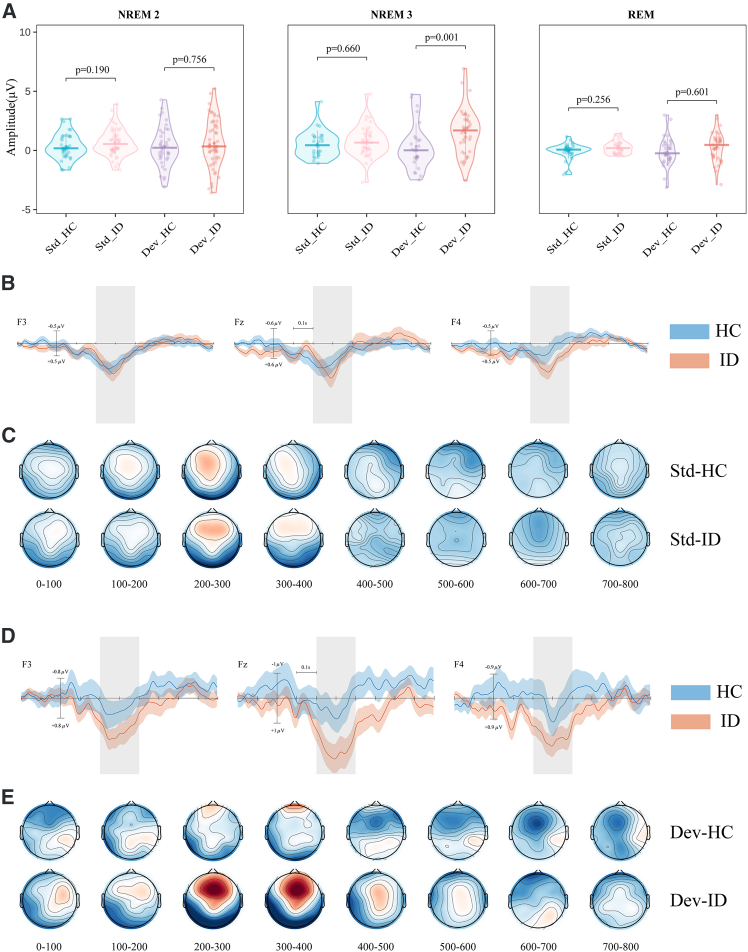
Event-related potentials and topographic maps in response to exogenous perturbation stimuli during sleep (A) P300 amplitude for the HC and ID groups across N2, N3, and REM sleep stages. For deviant stimuli during N3 sleep, ID group showed significantly larger P300 amplitudes than the HC group (ID: 1.33 ± 1.84; HC: 0.25 ± 1.51; HC − ID = −1.07, 95% CI [−1.69, −0.45], *t* = −3.40, *p* = 0.001, p_FDR = 0.004, Cohen’s d = −0.95). No significant group differences were found for standard stimuli in N3 sleep (HC − ID = −0.14, 95% CI [−0.76, 0.48], *t* = −0.44, *p* = 0.660, p_FDR = 0.756) or for either stimulus type in N2/REM sleep (all p_FDR > 0.05). (B and C) Representative ERP waveforms and scalp topography in response to standard stimuli during N3 sleep for HC (blue) and ID (red); scalp topography shows successive 100-ms time windows, with HC displaying low-amplitude, diffuse activity. (D and E) Representative ERP waveforms and scalp topography in response to deviant stimuli during N3 sleep for HC (blue) and ID (red); scalp topography shows successive 100-ms time windows, with ID displaying prominent anterior-positive clusters spanning 200–300 and 300–400 ms. P300 amplitudes were extracted from the 200–400 ms latency window over frontal electrodes (F3, F4, and Fz) and averaged to define a frontal ROI. Std and Dev denote standard and deviant stimuli, respectively. Shaded areas in ERP waveforms represent SEM. p_FDR denotes the Benjamini-Hochberg false discovery rate-adjusted *p* value.

**Figure 3:**
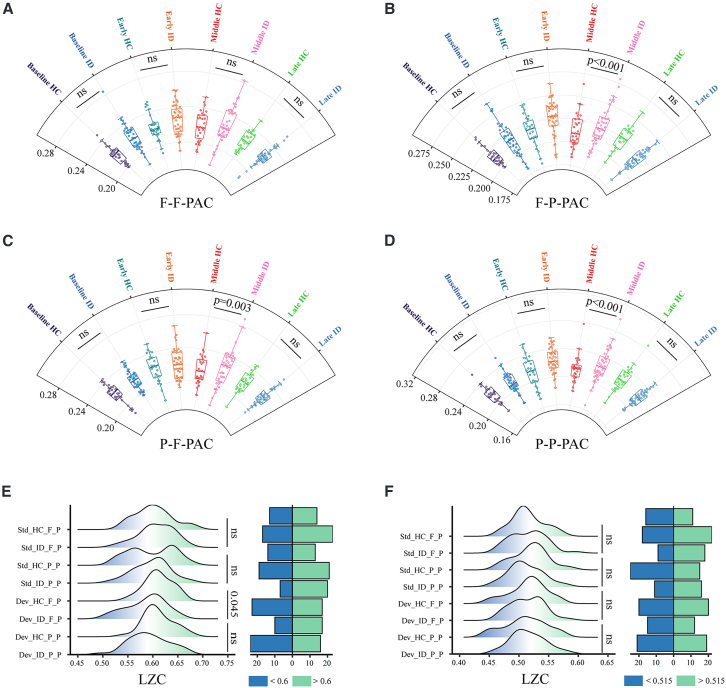
Altered cross-frequency coupling strength and coupling pattern complexity during N3 sleep under auditory perturbation (A–D) Power-controlled event-related phase-amplitude coupling (ERPAC) for four pathways (F-F, F-P, P-F, and P-P) across baseline, early, middle, and late windows under standard/deviant conditions. (A) F-F: No significant group difference survived FDR correction (p_FDR = 0.071). (B) F-P: ID group had significantly higher ERPAC than HCs in the middle window (200–400 ms) under deviant condition (HC − ID = −0.013, 95% CI [−0.020, −0.006], t = −3.67, *p* < 0.001, p_FDR = 0.003, Cohen’s d = −1.02). (C) P-F: ID group showed significantly elevated ERPAC compared with HCs in the middle window under deviant condition (HC − ID = −0.011, 95% CI [−0.018, −0.004], *t* = −2.96, *p* = 0.003, p_FDR = 0.016, Cohen’s d = −0.81). (D) P-P: ID group had significantly higher ERPAC than HCs in the middle window under deviant condition (HC − ID = −0.015, 95% CI [−0.022, −0.008], *t* = −4.24, *p* < 0.001, p_FDR <0.001, Cohen’s d = −1.17). No significant group differences were observed under standard condition. (E and F) Lempel-Ziv complexity (LZC) of ERPAC time series across the middle (200–400 ms) and late (400–800 ms) windows (F-P/P-P pathways as examples). (E) Middle window: Trend-level difference in F-P pathway under deviant condition (HC > ID, HC − ID = 0.015, 95% CI [0.0003, 0.030], *t* = 2.01, *p* = 0.045, p_FDR = 0.900, Cohen’s d = 0.59); no FDR-surviving effects. (F) Late window: No significant group differences in LZC were observed across any pathway/stimulus (all p_FDR > 0.05). ns, not significant. p_FDR denotes the Benjamini-Hochberg false discovery rate-adjusted *p* value.

To evaluate the evaluative neural responses to external stimuli during sleep, P300 amplitudes were extracted from the 200–400 ms latency window over frontal electrodes (F3, F4, and Fz) and averaged to define a frontal region of interest (ROI). Linear mixed-effects model analyses revealed a significant main effect of group (F(1, 93) = 4.86, *p* = 0.030, η_p_^2^ = 0.05), indicating overall larger P300 amplitudes in individuals with ID than in HCs. A significant main effect of sleep stage was also observed (F(2, 400) = 5.49, *p* = 0.004, η_p_^2^ = 0.027), demonstrating that P300 amplitude varied systematically as a function of sleep depth. In contrast, the main effect of stimulus type was not significant (F(1, 347) = 0.57, *p* = 0.449, η_p_^2^ = 0.002), and neither the group × stage (F(2, 400) = 1.14, *p* = 0.322, η_p_^2^ = 0.006) or group × stimulus (F(1, 347) = 0.53, *p* = 0.469, η_p_^2^ = 0.002) interactions nor the stage × stimulus (F(2, 347) = 0.46, *p* = 0.634, η_p_^2^ = 0.003) interactions reached statistical significance. Importantly, a significant three-way interaction among group, sleep stage, and stimulus type was detected (F(2, 347) = 3.09, *p* = 0.047, η_p_^2^ = 0.018), indicating that the effect of ID on stimulus processing was specific to both sleep stage and stimulus. Post hoc analyses further demonstrated that group differences were primarily driven by responses to deviant stimuli during deep sleep. Specifically, during N3 sleep, frontal P300 amplitudes elicited by deviant stimuli were significantly larger in the ID group than in HCs (ID: 1.33 ± 1.84; HC: 0.25 ± 1.51; HC− ID = −1.07, 95% CI [−1.69, −0.45], *t* = −3.40, *p* = 0.001, p_FDR = 0.004, Cohen’s d = −0.95; Figures 2A and 3D). In contrast, no significant group differences were observed either for standard stimuli during N3 sleep (HC − ID = −0.14, 95% CI [−0.76, 0.48], *t* = −0.44, *p* = 0.660, p_FDR = 0.756; Figures 2A and 3B) or for either stimulus type during N2 or REM sleep (all p_FDR >0.05, Figure 2A). This effect was further supported by scalp topography analyses: within 100 ms sliding windows, the ID group displayed prominent anterior-positive clusters spanning 200–300 ms and 300–400 ms, with a posterior-directed gradient. In contrast, the HC group showed only low-amplitude, diffuse topographic activity during these intervals (Figures 2C and 3E). No comparable differential activity was observed in other time windows. Collectively, these results are consistent with reduced resistance to external perturbations in patients with ID. Even within the same macro-scale N3 sleep stage, the ID group demonstrated heightened sensitivity to deviant stimuli, suggesting that, at a systems level, the ID-affected brain may be more susceptible to perturbation and more readily displaced from a deep-sleep attractor state.

### Containment: Cross-frequency gating and coupling pattern diversity

We next examined cross-frequency gating during N3 sleep to evaluate the integrity of neural containment-related processes, while further characterizing θ-β CFC strength after controlling for spectral power. At the global level, a significant main effect of group was observed (F(1, 74) = 6.89, *p* = 0.011, η_p_^2^ = 0.085), indicating an overall difference in θ-β coupling strength between the ID and HC groups when averaged across stimulus conditions, regional pathways, and time windows. The main effect of condition was also highly significant (F(1, 1545) = 34172.94, *p* < 0.001, η_p_^2^ = 0.957), showing that deviant stimuli elicited stronger θ-β coupling than standard stimuli in both groups. Significant group × condition (F(1, 1542) = 8.30, *p* = 0.004, η_p_^2^ = 0.005) and group × condition × time window interactions (F(2, 1541) = 8.15, *p* < 0.001, η_p_^2^ = 0.010) further indicated that ERPAC differences between the groups were temporally specific and dependent on the stimulus context. FDR-corrected post hoc comparisons showed that these group differences were concentrated in the middle latency window (200–400 ms) under the deviant condition. Contrasts were defined as HC minus ID, such that negative estimates indicate higher ERPAC values in the ID group. Within this time window, ERPAC was significantly elevated in the ID group relative to the HC group across the frontal→parietal (F-P), parietal→frontal (P-F), and parietal→parietal (P-P) pathways, specifically in the F-P pathway (HC − ID = −0.013, 95% CI [−0.020, −0.006], *t* = −3.67, *p* < 0.001, p_FDR = 0.003, Cohen’s d = −1.02, Figure 3B), the P-F pathway (HC − ID = −0.011, 95% CI [−0.018, −0.004], *t* = −2.96, *p* = 0.003, p_FDR = 0.016, Cohen’s d = −0.81, Figure 3C), and the P-P pathway (HC − ID = −0.015, 95% CI [−0.022, −0.008], *t* = −4.24, *p* < 0.001, p_FDR <0.001, Cohen’s d = −1.17, Figure 3D). By contrast, the group difference in the frontal→frontal (F-F) pathway did not survive FDR correction (p_FDR = 0.071, Figure 3A), and no significant between group effects were detected under the standard condition. Taken together, these findings show that enhanced θ-β CFC in ID is not a generalized phenomenon, but instead emerges selectively during a critical middle latency stage following deviant stimulation, even after controlling for spectral power.

To further characterize the dynamical complexity of CFC, we analyzed the LZC of the ERPAC time series within the same statistical modeling framework and included ERPAC strength as a covariate. No significant main effect of group was observed at the global level (F(1, 67) = 0.071, *p* = 0.790, η_p_^2^ = 0.001), indicating that the two groups did not differ reliably in the overall coupling pattern complexity. In contrast, LZC was significantly modulated by condition (F(1, 1595) = 208.41, *p* < 0.001, η_p_^2^ = 0.116), coupling mode (F(3, 1541) = 27.06, *p* < 0.001, η_p_^2^ = 0.050), and time window (F(2, 1541) = 1784.57, *p* < 0.001, η_p_^2^ = 0.698). The ERPAC strength covariate was also significant (F(1, 1597) = 208.85, *p* < 0.001, η_p_^2^ = 0.116), indicating that coupling pattern complexity was systematically shaped by stimulus condition, spatial pathway, and temporal stage. At the local-comparison level, during the 200–400 ms window under the deviant condition, the F-P pathway showed a group difference in the direction of higher LZC in HC than in ID group (HC − ID = 0.015, 95% CI [0.0003, 0.030], *t* = 2.01, *p* = 0.045, p_FDR = 0.900, Cohen’s d = 0.59, Figure 3E). The other pathways including P-F and P-P showed changes in a similar direction but did not reach statistical significance. Because none of these local effects survived FDR correction, they were more appropriately interpreted as trend-level signals rather than robust focal abnormalities. Taken together with the ERPAC findings, these trend-level LZC patterns may be consistent with reduced coupling pattern diversity in ID during the middle post-stimulus window, but they did not survive FDR correction and, therefore, should be interpreted cautiously.

### Recovery: Late-window convergence after perturbation

Finally, we examined the late processing window (400–800 ms) to assess post-perturbation recovery of neural dynamics. Using the same analytical parameters and modeling strategy, we compared ERPAC and LZC between the groups during this interval. No significant group differences were observed for either coupling strength or coupling pattern complexity across any coupling mode under either deviant or standard stimulus conditions (all p_FDR > 0.05, Figure 3F). Relative to the transient abnormalities observed during the 200–400 ms window, both ERPAC and LZC converged between the groups in the late window, with values in the ID group returning to levels comparable to those in HCs. These findings suggest that cross-frequency gating abnormalities in ID are transient and event locked rather than sustained and are consistent with preserved late-stage recovery and re-stabilization.

### Exploratory mediation

**Figure 4:**
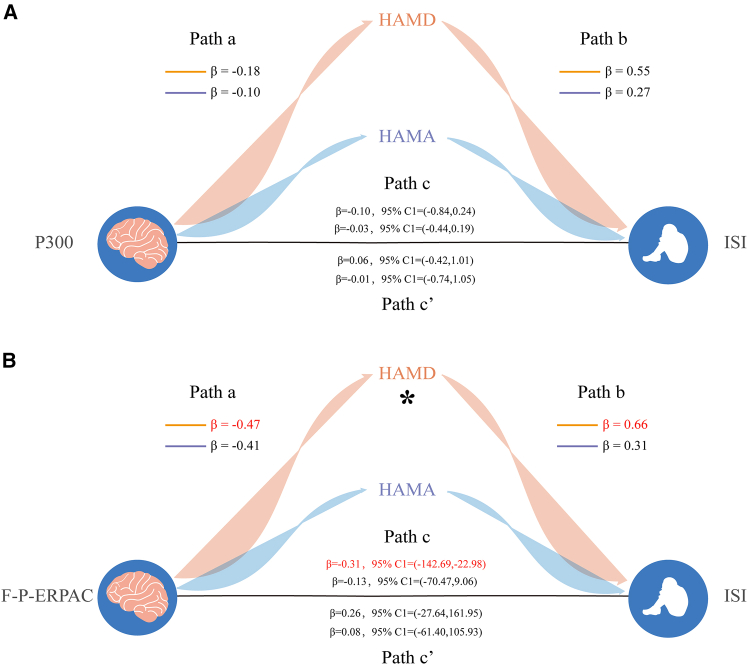
Mediation analysis of neurophysiological metrics on insomnia severity through anxiety and depression symptoms (A) P300 amplitude: No significant mediation pathways were observed (all *p* > 0.05). (B) F-P ERPAC: Significant indirect pathway from F-P ERPAC to ISI through HAMD (indirect effect: *p* = 0.023, B = −71.91, 95% CI [−142.69, −22.98], β = −0.31). ∗ denotes *p* < 0.05.

Given that convergent abnormalities across ERPAC strength and coupling pattern complexity were most robust for the F-P pathway, we next conducted an exploratory mediation analysis focusing on P300 and F-P ERPAC. Mediation modeling was performed using P300 and F-P ERPAC as predictors, HAMA and HAMD as mediators, and ISI as the outcome, while adjusting for age and sex. Among the tested models, only the indirect pathway from F-P ERPAC to ISI through HAMD reached statistical significance (indirect effect, *p* = 0.023, B = −71.91, 95% CI [−142.69, −22.98], β = −0.31; Figure 4B). Decomposition of the model showed that both constituent paths were significant. Specifically, F-P ERPAC was negatively associated with HAMD scores (*p* = 0.002, B = −122.92, 95% CI [−203.37, −44.89], β = −0.47), whereas higher HAMD scores predicted greater insomnia severity (*p* < 0.001, B = 0.59, 95% CI [0.26, 0.92], β = 0.66). After adjustment for HAMD, the direct effect of F-P ERPAC on insomnia severity was no longer significant (*p* = 0.208), and the total effect was likewise non-significant (*p* = 0.782). No other mediation pathways reached statistical significance (*p* > 0.05, Figure 4).

## Discussion

Our multi-stage nocturnal recordings identified N3 sleep as a sensitive detection window for impaired sleep resilience in ID. Patients exhibited a core triad of deficits specific to this stage: elevated evoked complexity, amplified frontal P300 to deviants, and enhanced fronto-parietal θ-β coupling, together with trend-level indications of reduced coupling pattern diversity during early post-stimulus processing. These early abnormalities, which reflect compromised resistance and containment, normalized in later time windows, demonstrating intact recovery. This N3-specific vulnerability establishes deep sleep as both a biomarker source and a therapeutic target, revealing a pathophysiology characterized by phasic gating failures, rather than sustained hyperarousal.^23^

The N3-specific rise in PE, irrespective of the stimulus category, indicates increased variability in post-stimulus neural dynamics during deep sleep in ID, suggesting a reduced constraint on evoked responses following external perturbation. This aligns with the concept of “local sleep” instability, where even during macrostructurally intact deep sleep,^24^ regional cortical excitability remains elevated in ID.^25^ Our findings suggest that such irregular evoked dynamics may be associated with heightened sensitivity to external perturbation, consistent with contemporary views of altered cortical excitability in ID.^26^ Furthermore, this heightened cortical sensitivity is reflected in the preservation of complex sensory discrimination during sleep, as evidenced by the ability to distinguish novel from familiar stimuli, particularly when inputs align with ongoing neural dynamics.^27^ The concomitant amplification of the frontal P300 to duration deviants provides an event-locked signature of exaggerated evaluative processing during deep sleep. Importantly, the frontal P300 observed here should not be equated with the canonical cognitive P300 typically described in wakefulness, but rather interpreted as a sleep state-dependent marker of salience evaluation or perturbation sensitivity under nocturnal auditory disturbance. This contrasts with the precisely coordinated slow oscillation-spindle coupling observed in healthy brains, which supports robust sensory gating and hippocampus-dependent memory consolidation during NREM sleep.^28^ Consequently, the heightened P300 response in ID, thus, reflects a failure to maintain the protective thalamocortical dynamics that normally restrict sensory processing during deep sleep. The selectivity to N3 and to deviant rather than standard events, therefore, points to a failure of state-dependent protection, rather than a generic elevation of arousal. Collectively, while conventional metrics might suggest normalized stability once N3 is reached,^29^^,^^30^ our results indicate that altered regulation of evoked neural dynamics persists during deep sleep in ID.

During the containment phase, the brain exhibits distinct oscillatory profiles that reflect its computational state. In resilient N3 networks, selective θ-β ERPAC increases in direction-specific interregional phase-amplitude dependency patterns involving F-P and P-P pairs are accompanied by a preservation of pattern diversity, as slow-frequency phases locally constrain fast-band power and restrict spatial propagation. This configuration, consistent with established θ-γ coupling mechanisms,^31^ enhances perturbation dissipation and supports multi-scale information integration. Conversely, our data indicate a deviation from this resilient state, characterized by enhanced fronto-parietal propagation accompanied by a nominal reduction in coupling pattern diversity, which did not survive correction for multiple comparisons. This observation aligns with systems-level models in which precise slow-fast timing and thalamocortical dynamics control gate cortical routing.^32^ This alteration in slow-phase gating may facilitate more widespread fast-power synchronization, potentially strengthening neural coupling while reducing coupling pattern variability. Such a gain-over-diversity trade-off may predispose the system toward more spatially extensive, wake-like patterns of activity.^33^ Importantly, the directionality of ERPAC described here reflects interregional phase-amplitude dependency rather than strict information flow, effective connectivity, or causal interaction.

During early neural processing, we observed marked impairments in neural disturbance resistance and containment mechanisms, which were subsequently followed by converging ERPAC profiles and coupling pattern diversity in later time windows. This trajectory reflects a progressive normalization of system dynamics, substantiating the notion of a phasic abnormality characterized by temporally delimited disruptions in neural gating, rather than a persistent pathological cascade. Our interpretation is consistent with evidence showing the sleeping brain’s preserved capacity to process external stimuli and extract behaviorally relevant information within discrete temporal intervals.^34^ Furthermore, we demonstrate that these functions are regulated by dynamic modulations in phasic gating mechanisms, which produce transient, temporally constrained perturbations without progressing to system-wide dysregulation. This framework aligns with the established neurophysiological principles wherein neural stability depends on a rapid return to slow-wave attractor states following perturbation. From a systems perspective, the brain under these conditions exhibits a dual characteristic: heightened vulnerability to destabilization coupled with a preserved capacity to reconstitute slow-wave organization within hundreds of milliseconds. This dynamic regulation effectively contains, without fully abolishing, the risk of progressive network fragmentation.

The identified triad of N3 abnormalities—elevated evoked complexity, amplified P300 responses, and altered fronto-parietal θ-β coupling with nominally reduced pattern diversity—suggests a link between macro-scale sleep instability and mesoscopic neural dynamics in ID. Our event-locked paradigm provides a quantifiable sleep resilience framework wherein neurophysiological metrics map onto specific dimensions: PE and P300 amplitude index disturbance resistance, while ERPAC combined with LZC quantifies containment integrity. This operationalization enables precise resilience assessment through deviation from healthy dynamic ranges across spatiotemporal domains. These metrics show promise for patient stratification and monitoring of targeted interventions such as phase-locked acoustic stimulation.^35^ Moreover, mediation analysis reveals that depressive symptoms partially translate impaired frontal-parietal containment into clinical severity, highlighting how compromised neural gating propagates through affective pathways.^36^ It should be noted that the total and direct effects of F-P ERPAC on insomnia severity were not significant, and, thus, these mediation results are exploratory and should be interpreted with caution. These findings support integrated treatment strategies addressing both thalamocortical gating and emotional regulation mechanisms.

### Limitations of the study

The study has several limitations. Spatial resolution is limited, and interpretations of fronto-parietal gating and thalamocortical dynamics are based on sensor-level EEG; so, references to thalamocortical mechanisms should be interpreted cautiously. The reliability of the resilience triad and its sensitivity to interventions require validation in larger cohorts a- multiple nights. Due to the stage-specific and relatively short recordings, functional sleep outcome measures such as stage transitions, micro-arousal rates, or cyclic alternating pattern indices could not be assessed. Future studies could address these issues by using source-localized or laminar recordings to improve anatomical resolution, employing longer continuous recordings to capture stage transitions and micro-arousals, and applying closed-loop stimulation to test causal effects on neural gating and sleep resilience.

## Resource availability

### Lead contact

Requests for further information, resources, and reagents should be directed to and will be fulfilled by the lead contact, Xiaoli Li (xiaoli@bnu.edu.cn).

### Materials availability

This study did not generate new unique reagents.

### Data and code availability

• All data reported in this paper will be shared by the lead contact upon request.
• All original code used for the analyses reported in this paper has been deposited at Zenodo: https://doi.org/10.5281/zenodo.20070128 and is publicly available as of the date of publication. The corresponding source repository is available at GitHub: https://github.com/guhai123/Impaired-sleep-resilience-underlies-transient-neural-instability-in-insomnia-disorder.
• Any additional information required to reanalyze the data reported in this paper is available from the lead contact upon request.

## Acknowledgments

This work was supported by the Brain Science and Brain-like Intelligence Technology-National Science and Technology Major Project under grant 2021ZD0204300, the Natural Science Foundation of Hubei Province (2025AFC109), the General Program of National Nature Science Foundation of China (62271331), and the Key Program of National Nature Science Foundation of Beijing (22Z30074).

## Author contributions

Conceptualization, C.Y., C.W., J.M., and X. Li; resources and data curation, J.M., G.C., X.S., X. Liu, Q.Y., and G.W.; methodology and formal analysis, C.Y., C.L., Hai Gu, S.W., H.C., and Heng Gu; writing – original draft, C.Y., P.G., J.Z., L.W., and R.W.; writing – review and editing, J.M., C.W., and X. Li. All authors have reviewed and approved the final version of the manuscript for publication.

## Declaration of interests

The authors declare no competing interests.

## Declaration of generative AI and AI-assisted technologies in the writing process

The authors take full responsibility for the content of this publication. During the preparation of this work, ChatGPT was used for language polishing; following the use of this tool, the authors reviewed and edited the content as necessary to ensure its accuracy and appropriateness.

## STAR★Methods

### Key resources table

**Table:** 

REAGENT or RESOURCE	SOURCE	IDENTIFIER
**Software and algorithms**		

Custom code	This paper	Zenodo: https://doi.org/10.5281/zenodo.20070128
MATLAB R2023a	MathWorks	https://ww2.mathworks.cn/products/matlab.html; RRID:SCR_001622
EEGLAB	Swartz Center for Computational Neuroscience	https://sccn.ucsd.edu/eeglab/; RRID:SCR_007292
PyCharm	JetBrains	https://www.jetbrains.com/pycharm/; RRID:SCR_018221
NeuroKit2	Makowski et al.^41^	https://github.com/neuropsychology/NeuroKit; RRID:SCR_021889
Tensorpac	Combrisson et al.^43^	https://github.com/EtienneCmb/tensorpac
RStudio	Posit	https://posit.co/products/open-source/rstudio; RRID: SCR_000432
R 4.5.0	R Core Team	https://www.r-project.org/; RRID:SCR_001905
Lavaan	Rosseel^46^	https://www.lavaan.ugent.be/; RRID:SCR_027663

### Experimental model and study participant details

For hypothesis testing, we conducted a multi-stage nocturnal study comparing ID patients to HC, implementing event-locked auditory perturbations during sleep verified through objective measures. A total of 43 HC participants and 58 patients with ID were enrolled in this study. The HC group included 16 males and 27 females, with a mean age of 40.81 ± 13.88 years, whereas the ID group included 14 males and 44 females, with a mean age of 45.71 ± 13.12 years. HC participants had self-reported healthy sleep patterns, characterized by ∼7–9 h of nocturnal sleep and no sleep disturbance history. ID participants were recruited from Wuhan First Hospital, all fulfilling DSM-5 criteria for ID.^37^ Strict exclusion criteria for the ID group (to minimize confounders): age outside 18–65 years; confirmed neurological, severe cardiovascular/cerebrovascular (e.g., stroke), liver/kidney failure, or severe mental illnesses (e.g., schizophrenia, bipolar disorder); comorbid primary sleep disorders (narcolepsy, OSAHS, PLMD); CNS-acting medication use (benzodiazepines, antidepressants) ≤14 days pre-enrollment or inability to discontinue during the study; or documented alcohol/nicotine dependence/substance abuse.

All participants completed standardized clinical assessments: Pittsburgh Sleep Quality Index (PSQI, overall sleep quality), Insomnia Severity Index (ISI, insomnia symptom severity), Mini-Mental State Examination (MMSE, basic cognitive function), Hamilton Anxiety Rating Scale (HAMA, anxiety levels), and Hamilton Depression Rating Scale (HAMD, depressive symptom severity). The study protocol was approved by the ethics committee of Wuhan First Hospital (WYIYLS [2022]22). Written informed consent was obtained from each participant prior to enrollment, and the study was conducted in strict accordance with the principles of the declaration of Helsinki.

### Method details

#### Overnight protocol with real-time manual staging (N2/N3/REM) and event-locked auditory perturbation

Preparation was scheduled to coincide with each participant’s habitual bedtime so that electrode setup finished immediately before lights-out. A certified sleep technologist continuously monitored the live recordings and manually staged 30-s epochs in real time across N2, N3, and REM according to AASM criteria. Stimulation blocks were initiated only after a stable target stage (N2/N3/REM) was verified and were terminated immediately upon any arousal or transition; only uninterrupted target-stage epochs were retained. The perturbation sequence consisted of standard tones (1000 Hz, 50 ms) interleaved with deviant tones (1000 Hz, 100 ms) delivered at 50 dB SPL with a fixed inter-stimulus interval of 600 ms.^27^^,^^38^ These stimulation parameters were selected to probe neural stability and sensory processing during sleep under mild exogenous perturbation, while minimizing the likelihood of overt arousal or substantial disruption of ongoing sleep.

### Quantification and statistical analysis

#### EEG acquisition and preprocessing

Continuous scalp activity was acquired using a 32-channel Grael amplifier set up in line with the 10–20 system, with horizontal electro-oculography recorded from the outer canthi. Electrode impedances were maintained under 5 kΩ, and all signals were digitized at a sampling rate of 1024 Hz. Pre-processing was conducted in EEGLAB^39^ running in MATLAB. Band-pass filtering (0.1–30 Hz) was applied to the data, which were then re-referenced to the global average, and screened for malfunctioning channels, which were excluded as needed. Ocular and myogenic artifacts were removed with extended independent component analysis. Trials were epoched −200 to 800 ms around stimulus onset and baseline-corrected (−200 to 0 ms).

#### Multidimensional assessment of sleep resilience

To operationalize the sleep resilience framework across its three core dimensions of disturbance resistance, containment, and recovery, we employed a set of complementary neurophysiological metrics derived from event-locked EEG responses. These metrics were designed to capture the brain’s dynamic reaction to perturbations at multiple temporal and functional scales.

#### Disturbance resistance: Evoked complexity and evaluative response

Disturbance resistance, reflecting the system’s inherent stability and its ability to minimize the initial impact of a perturbation, was assessed using two primary metrics: evoked neural complexity and the amplitude of the early evaluative neural response. PE^40^ was computed within the 0–800 ms post-stimulus window for each sleep stage (N2, N3, REM) and for each stimulus type (standard and deviant). Specifically, PE was first calculated separately for standard and deviant trials within each stage at the trial level, and trial-wise PE values were averaged per participant to form stimulus specific complexity estimates. These stimulus specific PE estimates were then averaged across stimulus types to obtain a single stage specific PE value per participant for the primary statistical analyses. PE essentially characterizes the probability distribution of ordinal patterns in a time series via phase-space reconstruction, followed by Shannon entropy computation. Formally, consider an EEG signal x = [x_1_,x_2_, …,x_N_] of length N, obtained from a single trial and a single channel. Given an embedding dimension m and a time delay τ, the embedding vectors are reconstructed as X_i_ = [x_i_,x_i+τ_, …,x_i+(m-1)τ_], i = 1, 2, …,N-(m-1)τ. For each embedded vector X_i_, the elements are sorted in ascending order to yield a corresponding permutation pattern π_i_. The set of all valid vectors defines the collection of permutation patterns. The relative frequency associated with each permutation pattern π_k_is then computed. If the k-th pattern occurs n_k_ times, its probability is given by *p*(*π*_*k*_) = *n*_*k*_/(*N*-(*m*-1)*τ*). The normalized PE is subsequently defined as:

$$PE=-\frac{1}{\log_{2}\left(m!\right)}\sum_{k=1}^{M}p\left(\pi_{k}\right)\log_{2}\;p\left(\pi_{k}\right)\text{,}$$

where *M* ≤ *m*! denotes the count of distinct patterns observed. In the present study, the embedding dimension was set to *m* = 3 and the time delay to *τ* = 1. The calculation of PE was carried out in the Python environment using the NeuroKit2 toolbox.^41^

For the event-related potential (ERP) indices, the P300 component was selected (across N2/N3/REM). The channels in the frontal lobe region were selected as the region of interest (ROI), and the average amplitude within the time window of 200–400 ms was extracted.

#### Containment: Cross frequency gating and pattern diversity

Containment, referring to the brain’s cross frequency gating-related response to perturbation-evoked activity, was evaluated through the strength and flexibility of cross frequency coupling mechanisms during the post-stimulus period. Event-related phase-amplitude coupling.^42^ (ERPAC) was computed between theta phase (4–8 Hz) and beta amplitude (13–30 Hz). Four *a priori* time windows were defined: a baseline window (−200 to 0 ms), an early window (0–200 ms), a middle window (200–400 ms), and a late window (400–800 ms). The middle window (200–400 ms) was designated *a priori* as the primary window for hypothesis testing, whereas the other windows were included to characterize temporal dynamics and recovery. ROI signals were derived from multi-channel montages (Frontal: FP1/FP2/F3/Fz/F4/F7/F8, Parietal: CP3/CPz/CP4/P3/Pz/P4). ERPAC was quantified for four PAC modes: Frontal→Frontal (F-F), Frontal→Parietal (F-P), Parietal→Frontal (P-F), and Parietal→Parietal (P-P). Coupling strength was estimated using the circular-linear correlation method, which quantifies trial-wise phase-amplitude alignment to assess time-resolved cross frequency coupling.^42^ For each time point t, phase frequency *f*_*p*_, and amplitude frequency *f*_*a*_, we test whether the high-frequency amplitude *A*_*k*_(*f*_*a*_,*t*) varies systematically with the low-frequency phase *ϕ*_*k*_(*f*_*p*_,*t*) across trials, as defined by:

$$ERPAC\left({f_{p},f}_{a},t\right)=\sqrt{\frac{r_{A\;\sin}^{\;2}+r_{A\;\cos}^{\;2}-2r_{A\;\sin}r_{A\;\cos\;}r_{\sin\;\cos}}{1-r_{\sin\;\cos}^{\;2}}}\text{,}$$

where r_A*sin*_ = corr(A_k_(f_a_,t),*sin*ϕ_k_(f_p_,t)), r_A*cos*_ = corr(A_k_(f_a_,t),*cos*ϕ_k_(f_p_,t)), r_*sincos*_ = corr(*sin*ϕ_k_(f_p_,t),*cos*ϕ_k_(f_p_,t)), and *k* indexes trials. ERPAC values approach 1 when amplitude shows a reliable dependence on phase across trials and approach 0 when amplitude is independent of phase. ERPAC was computed in Python using the Tensorpac toolbox.^43^

Coupling-pattern complexity was quantified as length-normalized Lempel-Ziv complexity (LZC) of the ERPAC time series within each *a priori* defined time window (baseline: −200 to 0 ms; early: 0–200 ms; middle: 200–400 ms; late: 400–800 ms) for every stimulus condition and coupling pathway. LZC indexes the diversity of temporal coupling patterns, with higher values indicating greater pattern diversity and lower values reflecting more stereotyped dynamics. For each window, continuous ERPAC signals were binarized using the within-window mean as the threshold, yielding a symbolic sequence *S* = *s*1,*s*2, …,*sn*, where *s*_*i*_∈{0,1}. The complexity *c* is then determined by sequentially scanning the sequence and attempting to identify matching substrings within the previously encountered portion. Whenever the current ‘look-ahead window’ substring cannot be found in the ‘search buffer’, it is identified as a new pattern, and the complexity counter is incremented. A detailed description of the algorithmic procedure can be found in the literature.^44^ Finally, the LZC value is normalized according to the following formula:

$$LZC=\frac{c\;\log_{2}\;n}{n}\text{,}$$

where c denotes the count of distinct substrings and *n* is the length of the symbolic sequence. This normalization aims to eliminate the effect of sequence length and thus enables comparison across signals. The calculation of LZC was carried out in the Python environment using the NeuroKit2 toolbox.^41^

#### Recovery: Late-window normalization

Recovery capacity was assessed by examining late-stage dynamics of the cross frequency gating-related metrics within the same analytical framework used for the containment analysis. Specifically, recovery was evaluated by comparing ERPAC and LZC values in the late window (400–800 ms) with those observed in the middle window (200–400 ms) to characterize post-perturbation dynamics across successive processing stages.

#### Statistical and mediation analyses

Statistical analyses were performed using linear mixed-effects models implemented in R (4.5.0). Subjects were included as random intercepts to account for repeated measures. Models were fitted using maximum likelihood estimation. PE was averaged across stimulus types and analyzed as a function of Group (ID, HC) and Sleep stage (N2, N3, REM). P300 amplitudes were analyzed using a factorial model including Group, Stage, and Stimulus type (standard, deviant). Power-controlled ERPAC was analyzed during N3 sleep using full-factorial models including Group, Condition, Coupling mode, and Time window. LZC of the ERPAC time series was analyzed using the same fixed-effects structure, with ERPAC entered as a covariate to examine coupling-pattern complexity independent of mean coupling strength. Post hoc contrasts were performed using estimated marginal means, with *p*-values adjusted for multiple comparisons using the Benjamini-Hochberg false discovery rate procedure. Type III analyses of variance were used to evaluate fixed effects, and partial eta-squared was reported where appropriate. All statistical tests were two-tailed, with significance set at *p* < 0.05.^45^

Exploratory mediation analyses were conducted to test whether affective symptom burden mediated the association between EEG-derived resilience-related measures and insomnia severity (ISI) within a SEM framework in R using the lavaan package.^46^ Candidate EEG predictors were selected based on the main neurophysiological findings and their theoretical relevance to disturbance resistance and cross frequency gating. P300 amplitude was examined as an index of disturbance resistance, whereas ERPAC-derived measures were tested as cross frequency gating-related predictors. Separate models were fit for each predictor and for each mediator (HAMD, HAMA), with age and sex as covariates. The path specification was m ∼ a·x + c1·age + c2·sex and y ∼ b·m + c′·x + d1·age + d2·sex, where x is the EEG readout, m is the mediator, and y is ISI. The indirect effect (a×b) and the total effect (c′ + a×b) were analyzed using bias-corrected percentile bootstrap confidence intervals, with n set to 5000, and significance was defined by intervals not crossing zero.
